# Factors associated with reduced infliximab exposure in the treatment of pediatric autoimmune disorders: a cross-sectional prospective convenience sampling study

**DOI:** 10.1186/s12969-021-00548-8

**Published:** 2021-05-01

**Authors:** Ryan S. Funk, Valentina Shakhnovich, Yu Kyoung Cho, Kishore Polireddy, Taina Jausurawong, Kyle Gress, Mara L. Becker

**Affiliations:** 1 Department of Pharmacy Practice, The University of Kansas Medical Center, 3901 Rainbow Blvd, MS 4047, Rm 6013, Kansas City, KS 66160 USA; 2 Department of Pediatrics, University of Missouri-Kansas City, Kansas City, MO USA; 3 Children’s Mercy Kansas City, Kansas City, MO USA; 4 Georgetown University School of Medicine, Washington, DC USA; 5 Department of Pediatrics, Duke University Hospital, Durham, NC USA

**Keywords:** Infliximab, Pharmacokinetics, Pediatrics, Juvenile idiopathic arthritis, Inflammatory bowel disease, Uveitis

## Abstract

**Background:**

Inadequate systemic exposure to infliximab (IFX) is associated with treatment failure. This work evaluated factors associated with reduced IFX exposure in children with autoimmune disorders requiring IFX therapy.

**Methods:**

In this single-center cross-sectional prospective study IFX trough concentrations and anti-drug antibodies (ADAs) were measured in serum from children diagnosed with inflammatory bowel disease (IBD) (*n* = 73), juvenile idiopathic arthritis (JIA) (*n* = 16), or uveitis (*n* = 8) receiving maintenance IFX infusions at an outpatient infusion clinic in a tertiary academic pediatric hospital. IFX concentrations in combination with population pharmacokinetic modeling were used to estimate IFX clearance. Patient demographic and clinical data were collected by chart review and evaluated for their relationship with IFX clearance.

**Results:**

IFX trough concentrations ranged from 0 to > 40 μg/mL and were 3-fold lower in children with IBD compared to children with JIA (*p* = 0.0002) or uveitis (*p* = 0.001). Children with IBD were found to receive lower IFX doses with longer dosing intervals, resulting in dose intensities (mg/kg/day) that were 2-fold lower compared to children with JIA (*p* = 0.0002) or uveitis (*p* = 0.02). Use of population pharmacokinetic analysis to normalize for variation in dosing practices demonstrated that increased IFX clearance was associated with ADA positivity (*p* = 0.004), male gender (*p* = 0.02), elevated erythrocyte sedimentation rate (ESR) (p = 0.02), elevated c-reactive protein (CRP) (*p* = 0.001), reduced serum albumin concentrations (*p* = 0.0005), and increased disease activity in JIA (*p* = 0.009) and IBD (*p* ≤ 0.08). No significant relationship between diagnosis and underlying differences in IFX clearance was observed. Multivariable analysis by covariate population pharmacokinetic modeling confirmed increased IFX clearance to be associated with anti-IFX antibody positivity, increased ESR, and reduced serum albumin concentrations.

**Conclusions:**

Enhanced IFX clearance is associated with immunogenicity and inflammatory burden across autoimmune disorders. Higher systemic IFX exposures observed in children with rheumatologic disorders are driven primarily by provider drug dose and interval selection, rather than differences in IFX pharmacokinetics across diagnoses. Despite maintenance IFX dosing at or above the standard recommended range for IBD (i.e., 5 mg/kg every 8 weeks), the dosing intensity used in the treatment of IBD is notably lower than dosing intensities used to treat JIA and uveitis, and may place some children with IBD at risk for suboptimal maintenance IFX exposures necessary for treatment response.

**Supplementary Information:**

The online version contains supplementary material available at 10.1186/s12969-021-00548-8.

## Background

The introduction of biological therapies, including infliximab (IFX), has drastically altered the treatment course of chronic autoimmune disorders affecting children [1]. IFX is a chimeric IgG1 monoclonal antibody targeting tumor necrosis factor-alpha (TNFα), a major pro-inflammatory cytokine implicated in the pathogenesis of inflammatory autoimmune disorders [2]. As the first monoclonal antibody approved for pediatric indications, IFX remains one of the most well studied biologic agents and is widely used, on and off-label, for the management of several chronic autoimmune disorders, including inflammatory bowel disease (IBD) [3], juvenile idiopathic arthritis (JIA) [4, 5], and uveitis [6–8].

Although IFX has demonstrated efficacy in the treatment of these pediatric autoimmune disorders, clinical response is variable. Studies in IBD and rheumatoid arthritis (RA) indicate that 30% of patients fail to respond to IFX therapy and up to 50% of those who do respond lose response by 1 year of treatment [9, 10]. Inadequate drug exposure, determined by measuring serum trough concentrations of IFX, has been identified as a major source of primary therapeutic non-response or secondary loss of response to IFX in IBD [11]. Moreover, inadequate drug exposure resulting in ongoing disease activity in any condition can have long term and significant consequences (e.g., increased disease morbidity, decreased quality of life). IFX is currently not labeled for use in children with JIA, due in part to the drug not achieving the primary endpoint in a pivotal phase 3 placebo-controlled randomized-controlled trial, in which the dosing strategy, guided by adult dosing parameters, ultimately failed to result in similar drug exposures in children [12, 13].

Immunogenicity, the development of anti-drug antibodies (ADAs), is another major contributing factor to the pharmacokinetics of IFX and is believed to result in accelerated systemic clearance of IFX through immune-complex formation, resulting in risk of response failure [14]. In addition to immunogenicity, a number of other patient factors, including: gender, systemic inflammatory burden, serum albumin concentrations, concomitant therapy with immunomodulators, and body weight are thought to modulate IFX clearance and serum trough concentrations [15, 16]. Previous study results suggest that the pharmacokinetic properties of IFX vary by age resulting in increased clearance in the pediatric population [17, 18]. Similarly, population-based pharmacokinetic studies have found that the pharmacokinetics of IFX vary by diagnosis [19]. Anecdotally, in our experience, the prevalence and clinical concern for immunogenicity appear higher in children with IBD compared to patients treated for rheumatologic disorders. Therefore, the objective of this study was to compare IFX pharmacokinetics in children receiving IFX for the management of inflammatory autoimmune disorders and evaluate relevant sources of interindividual variability in real-world clinical practice, as the first step toward implementing precision therapeutics for IFX treatment of immuno-inflammatory disorders in children.

## Methods

### Study design and patients

This was a prospective cross-sectional study that collected blood samples and clinical data from a cohort of pediatric patients (*n* = 97) receiving maintenance IFX infusions at the Children’s Mercy Kansas City Infusion Center. Blood samples were collected from patients on stable IFX dosing (e.g., no changes in dose or interval for ≥2 dosing cycles), immediately prior to their scheduled infusion (i.e., trough samples). Blood samples were processed immediately, and the resulting serum aliquots were stored at − 80 °C prior to batch analysis. Relevant clinical and demographic data were collected from review of the electronic medical record. Information collected and used in these analyses included patient age, gender, diagnosis, IFX dose, prescribed dosing interval, time since last IFX infusion, concomitant disease modifying anti-rheumatic drug (DMARD) treatment with methotrexate (MTX) or azathioprine (AZA), most recent laboratory measurements of erythrocyte sedimentation rate (ESR), serum albumin concentrations, and C-reactive protein (CRP) levels, 71-joint count clinical juvenile arthritis disease activity scores (cJADAS-71), and IBD physician global assessment of disease activity (PGA) within 30 days of study visit, if available. cJADAS-71 was calculated as the sum of the physician global assessment (visual analog scale range of 0-10), the parent/patient global assessment (visual analog scale range of 0-10), and the active joint count (simple count range of 0-71). Clinical and laboratory parameters of IBD were assessed using PGA, with disease activity assigned as quiescent, mild, moderate, or severe via agreement by two independent pediatric gastroenterologists. Due to small sample size, severe (*n* = 1) and moderate (*n* = 7) IBD were combined and treated as a single disease activity group (moderate/severe) in subsequent statistical analyses. IFX dose intensity (mg/kg/d) was calculated as the average daily dose equivalent of IFX by dividing the patient’s weight-based IFX dose (mg/kg) by the dosing interval, represented by the number of days since the last IFX dose. The study was approved under the Children’s Mercy Kansas City institutional review board. Written informed consent/assent was acquired prior to inclusion of subjects in the study and collection of patient data and samples.

### IFX and anti-IFX analysis

Serum samples were submitted for analysis to ARUP Laboratories (Salt Lake City, UT) and IFX and anti-IFX antibodies were detected using a NF-ƙβ luciferase gene-reporter assay (GRA) [20]. The lower limit of IFX quantitation for the assay was 0.65 μg/mL and the upper limit of quantitation was 40 μg/mL. Serum IFX concentrations below the limits of quantitation were reported as 0 μg/mL and samples measuring above the limit of quantitation were reported as 40 μg/mL. Anti-IFX antibody detection was reported as positive or negative based on an infliximab neutralizing titer of 1:20 or greater. Anti-IFX antibodies in serum were additionally assessed using a commercial enzyme-linked immunosorbent assay (ELISA) in combination with an acid dissociation step following the manufacturer’s protocol (Eagle Biosciences, Amherst, NH). Anti-IFX antibody levels were reported in arbitrary units/mL. Samples with signal greater than two times background were deemed positive for anti-IFX antibodies.

### IFX clearance estimation by population pharmacokinetic modeling

Estimates for pediatric pharmacokinetic parameters for IFX were obtained from a population pharmacokinetic model developed from 112 children from the phase 3 REACH study and were used to estimate IFX clearance (Cl) in our patients [21]. Pharmacokinetic estimates for a typical child were as follows: clearance (Cl), 5.43 mL/kg/d; volume of distribution in the central compartment (V_1_), 54.2 mL/kg; volume of distribution in the peripheral compartment (V_2_), 29.2 mL/kg; intercompartmental clearance (Q), 3.52 mL/kg/d. A 2-compartment model with a 2-h intravenous infusion and first order elimination was used to estimate Cl of the IFX for each patient using a nonlinear mixed-effects approach in MONOLIX (Lixoft, Antony, France) [22]. Interindividual variability of Cl was evaluated using an exponential random effects model. The intraindividual variability was described as an additive residual error model. Undetected IFX concentration in four patients was substituted to half of the lower limit of quantification (i.e. 0.325 μg/mL).

### Covariate analysis

Variables associated with interindividual variability in Cl estimates were identified by population pharmacokinetic covariate analysis in MONOLIX. While IFX trough concentrations were measured in 97 patients, 15 patients were excluded due to missing covariates, and 82 patients were used to conduct covariate pharmacokinetic modeling. The covariates having significant influence were added in a stepwise manner with forward addition and backward elimination [23]. The covariate model was evaluated by the difference in the objective function value (ΔOFV), such that ΔOFV greater than 3.84 (*p* < 0.05, degree of freedom = 1) in forward addition and 7.88 (*p* < 0.005, degree of freedom = 1) in backward elimination, was indicative of significance using the log likelihood ratio test.

### Statistical analysis

Unpaired grouped analyses were conducted by Wilcoxon rank-sum testing. Spearman’s rank correlation analysis was used to evaluate correlations between continuous variables. Data analysis and statistical testing was conducted using JMP software v11 (SAS Institute Inc., Cary, NC). Statistical significance is considered for *p* < 0.05.

## Results

### Patient characteristics

Ninety-seven patients receiving maintenance IFX infusions, with a stable drug dose and dosing interval over the last two dosing periods, were enrolled in the study (Table 1). All participants gave their informed consent prior to inclusion in the study. The patient population consisted of three primary disease groups: IBD (*n* = 73), JIA (*n* = 16), and uveitis (*n* = 8). Dosing of IFX for patients with IBD was managed by pediatric gastroenterologists, while dosing for JIA and uveitis was managed by pediatric rheumatologists, as per standard of medical care at the providers’ discretion. Median patient age was 16 years and ranged from 5 to 21 years. Concomitant immunomodulatory DMARDs, either MTX or AZA, were used in 30% of children with IBD, 75% of children with JIA, and 75% of children with uveitis. Females represented 44% of the total patient population and varied by diagnosis, representing 40, 63, and 50% of IBD, JIA, and uveitis patients, respectively. The median prescribed IFX dose was 8.4 mg/kg (range 4.6 to 18.4 mg/kg), with a median prescribed dosing interval of 6 weeks (range 4 to 8 weeks), across the study population.

**Table 1:** Patient demographics and clinical data

Patient Characteristics	Study Population	IBD	JIA	Uveitis
Patients, *n*	97	73	16	8
Gender, *female, n (%)*	43 (44)	29 (40)	10 (63)	4 (50)
Age, *yr, median [range]*	16 [5,21]	16 [5,21]	13 [7,20]	16 [6,21]
Diagnosis, *n (%)*				
IBD	73 (75)			
JIA	16 (17)			
Uveitis	8 (8)			
IFX Dosing, *median [IQR]*				
Dose, *mg/kg*	8.4 [6.5,9.8]	7.7 [6.2,9.4]	9.7 [8.5,10.7]*	10.5 [7.5,12.0]^#^
Interval, *weeks*	6 [4,7]	6 [4,8]	4 [4,5]*	4 [4,6]^#^
Immunomodulator, *n (%)*				
MTX	37 (38)	19 (26)	12 (75)*	6 (75)^#^
AZA	3 (3)	3 (4)	0 (0)	0 (0)
Inflammatory Markers, *median [IQR]*				
ESR, *mm/hr*	9 [6,17.5]	9 [7,17]	7 [6,17]	13 [7,22]
CRP, *mg/dL*	0.5 [0.5,0.8]	0.5 [0.5,0.8]	0.5 [0.5,0.6]	0.7 [0.6,0.8]
Albumin, *g/dL*	4.3 [4.0,4.5]	4.3 [4.0,4.5]	4.3 [4.2,4.6]	4.3 [4.0,4.6]

*, IBD vs JIA, significant at *p* < 0.05

#, IBD vs Uveitis, significant at *p* < 0.05

### Variability in IFX trough levels

**Fig. 1:**
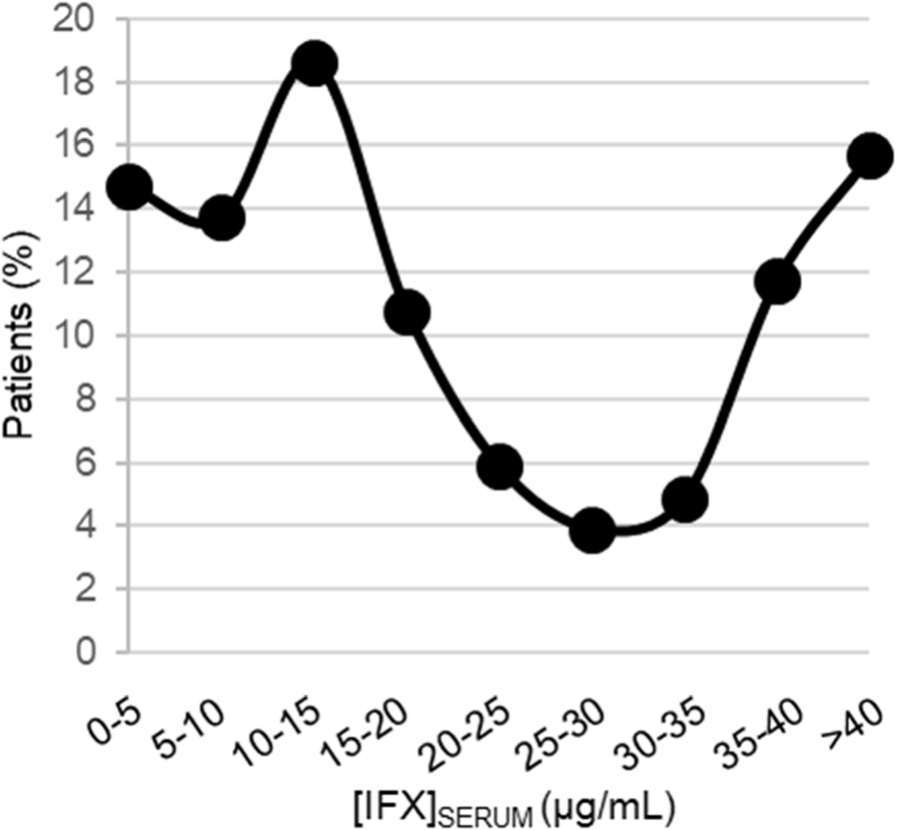
Distribution of serum trough concentrations of IFX in a cohort of pediatric autoimmune disorder patients receiving maintenance IFX infusions. The resulting histogram displays the percentage of patients in the experimental cohort (*n* = 97) with trough serum IFX concentrations in the ranges indicated

Median [IQR] IFX trough serum concentrations were 15.1 [7.2,34.7] μg/mL and spanned the limits of quantitation of the clinical assay, with levels below 0.65 μg/mL in 4 patients, and levels greater than 40 μg/mL in 16 patients. A histogram of the trough concentrations demonstrates a triphasic distribution with the majority of patients in the 10 to 15 μg/mL range, with secondary peaks representing patients with concentrations either less than 5 μg/mL, or greater than 40 μg/mL (Fig. 1). Trough levels below 5 μg/mL were measured in 15% of patients, and levels below 10 μg/mL in 28% of patients.

ADAs by the GRA were only found in three patients, all with non-detectable IFX trough concentrations resulting in median [IQR] IFX trough levels significantly lower than patients testing negative for ADAs by GRA (0 [0,0] vs 15.4 [7.8,35.0] μg/mL, *p* = 0.004). In addition to the detection of ADAs by GRA, patient samples were probed using a drug-tolerant immunoassay for ADAs and yielded detectable antibodies in a total of 9 patients (9.3% of study population), including the three detected by GRA and an additional 6 patients. Patients positive for ADA by immunoassay were also found to have significantly lower median [IQR] IFX trough levels compared to patients testing negative (3.2 [0,10.7] vs 15.8 [10.0,35.4] μg/mL, *p* = 0.0006). Similarly, measured anti-IFX ADA concentrations by immunoassay were associated with lower IFX trough levels (ρ = − 0.46, *p* < 0.0001). Compared to patients not concomitantly prescribed a DMARD, those receiving DMARD therapy in the form of MTX or AZA were found to have a lower incidence of ADA positivity by both GRA (3.5% vs 2.5%, *p* = 0.63) and by immunoassay (12.3% vs. 5.1%, *p* = 0.21), however, the differences lacked statistical significance.

### IFX trough level variation by diagnosis and dose intensity

**Fig. 2:**
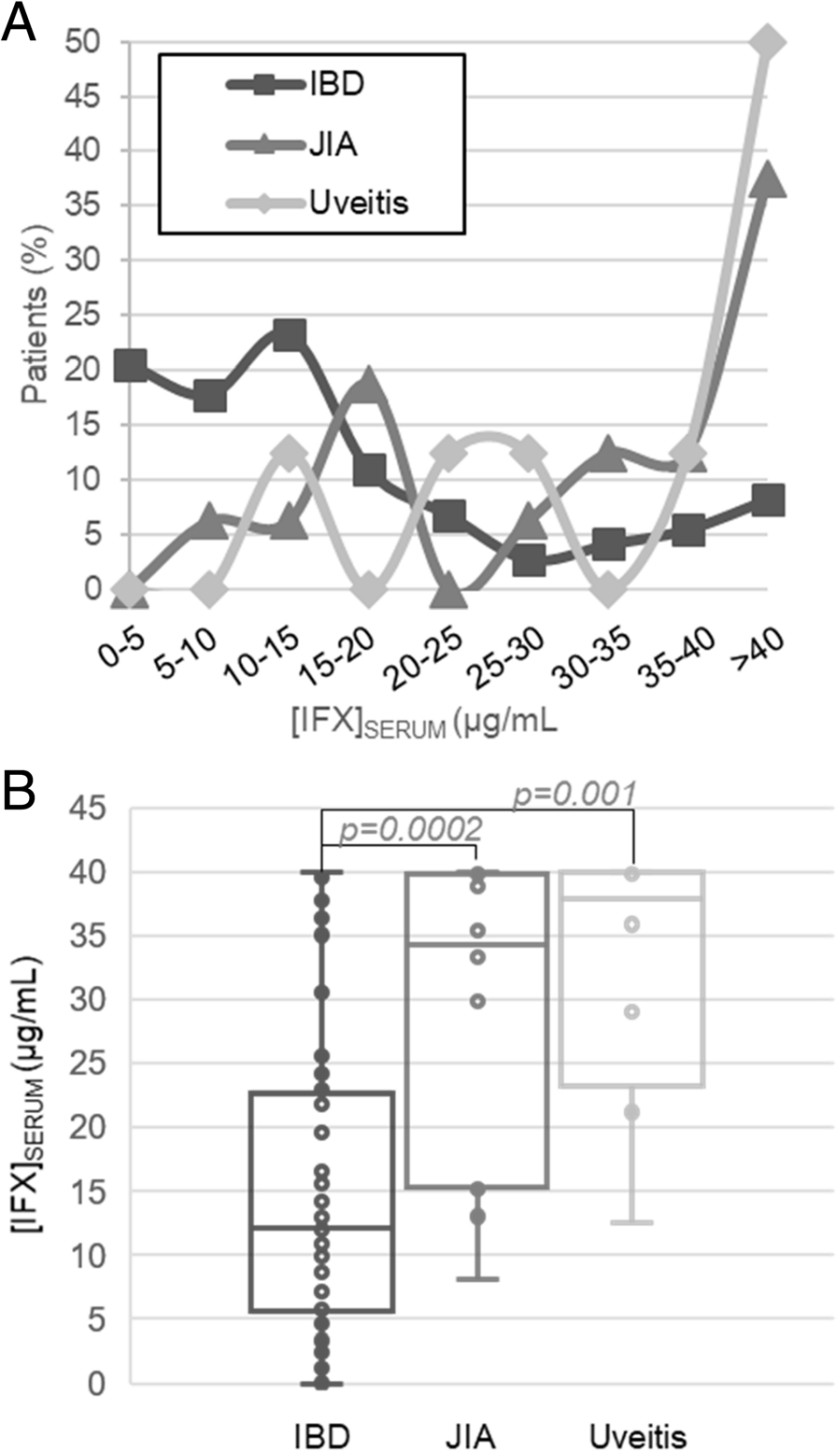
Serum trough concentrations of IFX based on diagnosis. **a** Serum trough IFX concentrations are presented as a histogram that displays the percentage of patients from the experimental cohort diagnosed with IBD (*n* = 73), JIA (*n* = 16), or uveitis (*n* = 8) with trough serum IFX concentrations in the indicated ranges. **b** IFX serum trough concentrations stratified based on diagnosis plotted as box and whisker plots and evaluated by unpaired analysis using the Wilcoxon rank-sum test and the resulting *p*-values are provided

Evaluation of IFX concentrations based on patient characteristics revealed that diagnosis was strongly associated with measured IFX trough levels (Fig. 2). Stratified by diagnosis, the histogram of IFX trough levels demonstrates that 21% of IBD patients and no patients with JIA or uveitis had levels < 5 μg/mL (Fig. 2a). Meanwhile, 50% of the uveitis patients, 38% of JIA patients, and only 8% of IBD patients had trough IFX levels > 40 μg/mL. IFX trough levels by diagnosis showed that children with JIA or uveitis had median [IQR] trough IFX concentrations three times greater than those being treated for IBD (Fig. 2b).

**Fig. 3:**
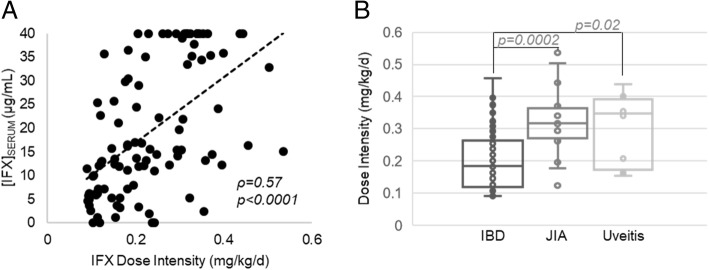
The association between IFX dose intensity and serum trough concentrations based on diagnosis. Dose intensity was determined as the average weight adjusted dose of IFX (i.e. mg/kg) per day, based on the timing between the last dose of IFX and the time at which the serum trough IFX concentrations were measured. **a** The association of IFX dose intensity and serum trough concentrations of IFX were evaluated by Spearman’s correlation analysis. The Spearman’s correlation coefficient (ρ) and associated *p*-value are provided. **b** Dose intensity stratified based on patient diagnosis is presented as a box and whisker plot and evaluated by unpaired analysis using the Wilcoxon rank-sum test and the resulting *p*-values are provided

Variation in dosing practices occurs between diseases and was investigated as a source for the observed variation in IFX trough levels by diagnosis. The combination of variation in both dose and dosing interval is best described as dosing intensity, which is the average daily dose equivalent of IFX in mg/kg/day determined by dividing the dose by the dosing interval. Increased dose intensity was found to be strongly associated with higher IFX trough levels (Fig. 3a). Components of the calculated dose intensity, both increased IFX dose (ρ = 0.43, *p* < 0.0001) and a shorter dosing interval, measured as the days since last dose (ρ = − 0.53, p < 0.0001), were also associated with increased trough IFX levels.

IFX dose intensity was stratified by diagnosis and demonstrated approximately 2-fold higher median [IQR] dose intensities in children with JIA (0.32 [0.27,0.36] mg/kg/d) or uveitis (0.35 [0.17,0.39] mg/kg/d) compared to children with IBD (0.18 [0.12,0.26] mg/kg/d), as shown in Fig. 3b. Children with IBD received lower median [IQR] IFX doses (7.7 [6.2,9.4] mg/kg) compared to children with JIA (9.7 [8.5,10.8] mg/kg, *p* = 0.0005), or uveitis (10.5 [7.5,12.0] mg/kg, *p* = 0.03). Similarly, physician prescribed dosing intervals were significantly longer in children with IBD, with median [IQR] dosing intervals of 6 [4,8] weeks in patients with IBD compared to 4 [4,4.8] weeks (*p* = 0.0002) for patients with JIA and 4 [4, 5.5] weeks (p = 0.03) for patients with uveitis. The relationship between dose intensity and ADA positivity was also explored and median [IQR] dosing intensities were observed to be lower in ADA positive patients compared to ADA negative patients by both GRA (0.12 [0.10,0.24] vs 0.21 [0.15,0.32] mg/kg/d, *p* = 0.19) and immunoassay (0.12 [0.10,0.28] vs 0.22 [0.15,0.32] mg/kg/d, *p* = 0.09), but were not found to reach statistical significance.

### Estimation of variability in IFX clearance

Recognizing variation in IFX dose and dosing interval are major covariates contributing to the observed variability in IFX trough levels in our patient population, the standard approach to controlling for differences in dose and dosing interval and evaluating covariates impacting drug disposition is the use of population pharmacokinetic modeling. Therefore, established population pharmacokinetic parameters were used in conjunction with measured trough IFX levels and patient dosing data to estimate IFX clearance in our patients and probe for covariates associated with the observed variation in estimated clearance [21]. Using a previously established two-compartment pharmacokinetic model we were able to obtain population clearance estimates. The mean estimate of population IFX clearance in the resulting structural pharmacokinetic model was 4.44 mL/kg/d (4.92% relative standard error).

**Fig. 4:**
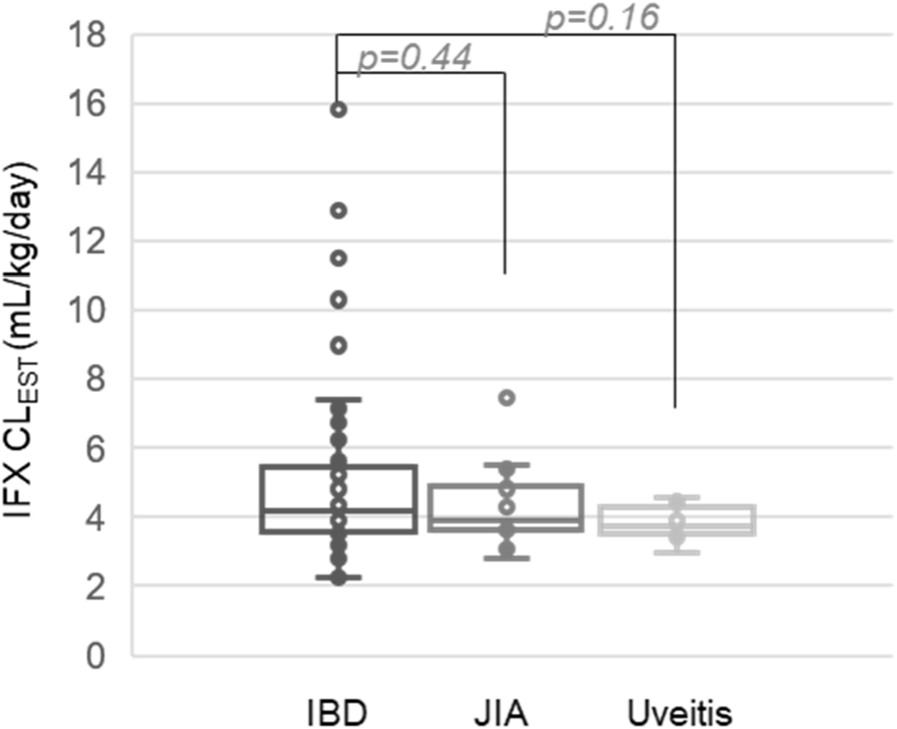
IFX clearance estimation and patient diagnosis. Estimated IFX clearance values stratified based on patient diagnosis are presented as a box and whisker plot and evaluated by unpaired analysis using the Wilcoxon rank-sum test and the resulting p-values are provided

IFX clearance values determined using the population model were compared based on diagnosis (Fig. 4). As illustrated, no significant association or trends were observed between diagnosis and IFX clearance. By univariate statistical analysis, increased IFX clearance was associated with increased ESR (ρ = 0.24,*p* = 0.02), increased CRP (ρ = 0.35,*p* = 0.001), increased ADA levels by ELISA (ρ = 0.22,*p* = 0.03) and reduced serum albumin concentrations (ρ = − 0.35,*p* = 0.0005), but failed to demonstrate an association with either age (ρ = − 0.09,*p* = 0.43) or weight (ρ = − 0.10,*p* = 0.31). Patient ADA positivity by GRA (*p* = 0.004) and male gender (p = 0.02) were also found to be associated with increased IFX clearance. However, no significant differences in IFX clearance were found based on DMARD use (*p* = 0.16), either with MTX (*p* = 0.30) or AZA (*p* = 0.29), or by ADA positivity by ELISA (*p* = 0.07).

**Fig. 5:**
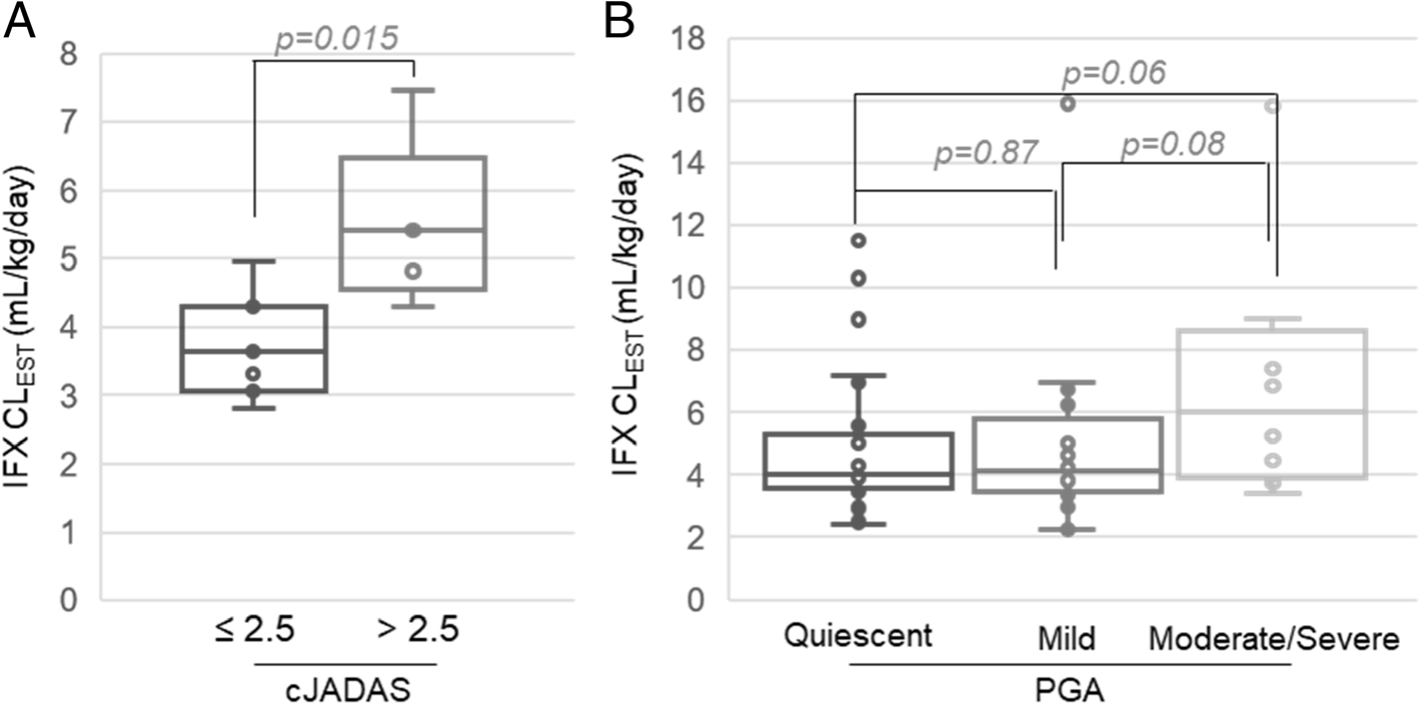
Association between estimated IFX clearance and disease activity. Estimated IFX clearance values are stratified and presented based on disease activity by (**a**) cJADAS-71 for patients with JIA and (**b**) by PGA for patients with IBD. Data is plotted as box and whisker plots and evaluated by unpaired analysis using the Wilcoxon rank-sum test and the resulting p-values are provided

Disease activity data for IBD (*n* = 74) and JIA (*n* = 12) were also collected when available and separately explored for associations with estimated IFX clearance. In children with JIA, an increased cJADAS-71 was found to be associated with increased IFX clearance (ρ = 0.71, *p* = 0.009). Stratification of the JIA population into moderate/high disease activity (i.e. cJADAS-71 > 2.5 across both oligo and polyarticular subtypes) and low disease activity (i.e. cJADAS-71 ≤ 2.5) showed that children with moderate/high disease activity had a 49% increase in IFX clearance compared to children with low disease activity (Fig. 5a). A comparison in children with IBD demonstrated a similar trend towards increased IFX clearance in more severe disease determined by PGA that was approaching statistical significance and resulted in a 50 and 47% increase in IFX clearance in children with moderate/severe disease compared to those with quiescent or mild disease, respectively (Fig. 5b).

**Fig. 6:**
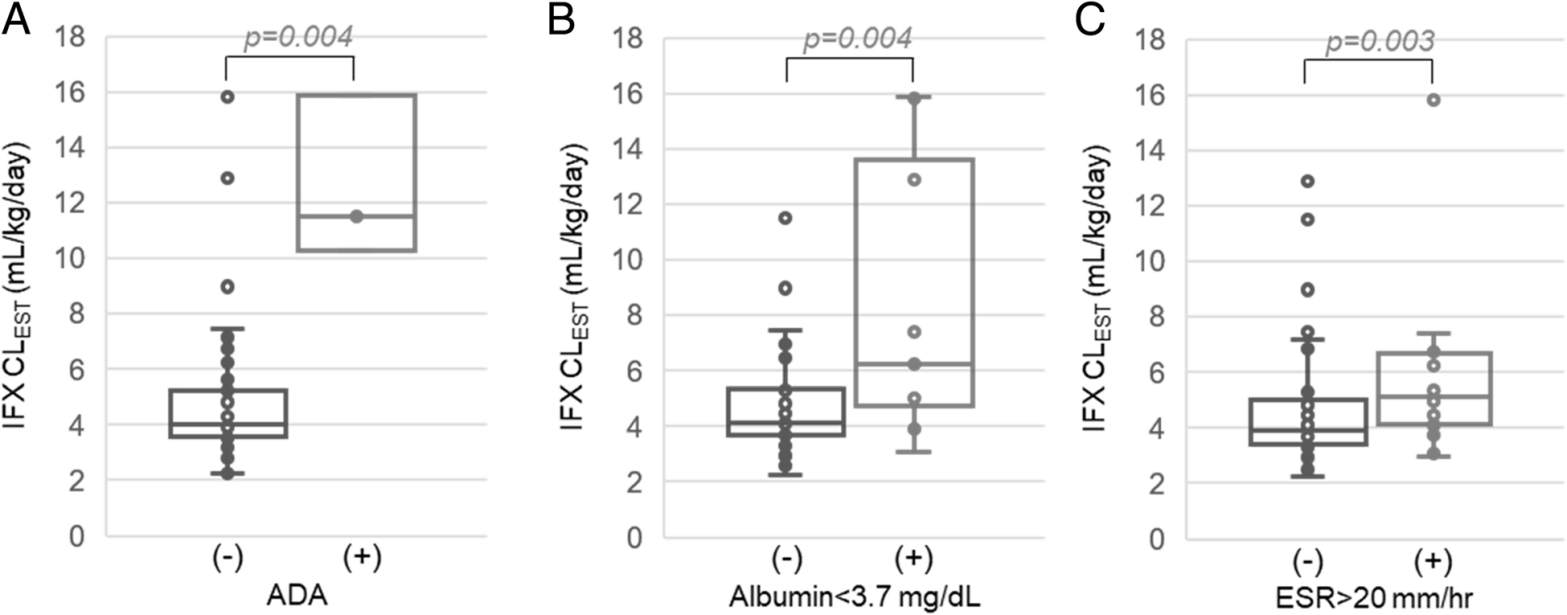
Association between estimated IFX clearance and identified clinical covariates. Estimated IFX clearance values are stratified and presented based on (**a**) ADA positivity by GRA, (**b**) serum albumin concentrations, and (**c**) ESR. Data is plotted as box and whisker plots and evaluated by unpaired analysis using the Wilcoxon rank-sum test and the resulting p-values are provided

A covariate pharmacokinetic model was also developed using a stepwise selection procedure as a multivariable statistical tool to identify patient variables associated with clearance (Supplementary Table 2). In this analysis, ADA positivity by GRA, serum albumin concentrations, and ESR were identified as significant covariates. The resulting equation describing the relationship between individual IFX clearance and the covariates in the final model are provided in Supplementary Table 3. The plots of the observed concentrations versus individual predicted concentrations and the individual predicted residuals are also provided as part of the goodness-of-fit evaluation in Supplementary Figure 1. IFX clearance values were stratified and plotted based on the variables identified in the covariate pharmacokinetic model (Fig. 6). Based on the univariate comparisons, anti-IFX antibody positivity by GRA was associated with a 286% increase in IFX clearance, serum albumin concentrations below 3.7 mg/dL were associated with a 54% increase in IFX clearance, and an ESR greater than 20 mm/hr was associated with a 31% increase in estimated IFX clearance.

## Discussion

Despite being a relatively well-studied biologic therapy, there remain significant gaps in knowledge regarding interindividual variability in IFX exposure, and the factors driving it in real-world practice. Even though IFX under-exposure is recognized as a major cause of primary drug non-response and secondary loss of response in pediatric patients, few pediatric studies have focused on understanding additional sources of interindividual variability in IFX exposure [17, 18, 24]. Ours is the first study to directly compare factors contributing to variability in IFX exposure in a diverse pediatric population, across three different diagnoses, with incorporation of population pharmacokinetic modeling to control for observed variation in dosing practices in real-world patient care.

Across the study population (*n* = 97), IFX dose intensity (i.e., dose and dosing interval) correlated strongly and positively with IFX trough levels (*p* < 0.0001). Only patients with undetectable IFX trough levels had ADAs detected by GRA and may reflect the lack of tolerance of this assay to detect ADA in the presence of excess free infliximab. Six additional patients were identified to have ADAs by immunoassay, highlighting differences in sensitivity across assay types used in clinical practice. All ADA positive patients (9% of study population, all with IBD) had significantly lower IFX trough levels than patients without ADAs, putting them at increased risk for therapeutic failure and increased risk of infusion reactions to IFX, which represents one of the only two biological agents approved for pediatric IBD.

Overall, IFX trough levels were significantly lower in pediatric patients with IBD, compared to JIA or uveitis (Fig. 2b; *p* ≤ 0.001); however, this is likely due to the lower dose intensity used in IBD, represented by both lower doses and longer dosing intervals. Population pharmacokinetic modeling confirmed that once we accounted for variability in dosing practices, IFX clearance rates were comparable across the three pediatric diagnoses investigated (Fig. 4). Nevertheless, the observed variability in dosing practices across diagnoses is important and offers an opportunity to compare and optimize practices across subspecialties. Looking at current dosing practices, 21 % of children with IBD were found to have IFX trough concentrations below the recommended trough levels associated with mucosal healing of IBD based on previous studies (i.e., < 5 μg/mL) [24]. Furthermore, only patients with IBD had detectable ADAs in our cohort, begging the question whether the real-world dosing practices observed in our IBD cohort (median [IQR] of 7.7 [6.2,9.4] mg/kg every 6 [4,8] weeks) are adequate to prevent loss of treatment response to IFX. Two other recent analyses have similarly questioned the adequacy of standard 5 mg/kg IFX dosing every 8 weeks for pediatric IBD, advocating for treat-to-target approaches for IFX [25, 26]. No patients with uveitis or JIA had ADAs or trough levels < 5 μg/mL, however, unlike IBD, there are no established IFX therapeutic trough targets currently utilized for JIA or uveitis. One may speculate that IFX troughs for rheumatologic conditions may need to be higher, based on evidence to suggest that systemic exposure may be inadequate to achieve acceptable drug concentrations at the target tissue of interest (e.g. the joint), which has resulted in trials utilizing direct intra-articular injections with IFX, or other biologic agents [27, 28]. Failure to observe ADAs in patients with JIA or uveitis in our study may indicate a reduced propensity for ADA formation in this patient population, or is more likely to be related to the maintenance of higher trough concentrations [29]. An alternative explanation may be that higher cumulative IFX dosing in JIA and uveitis may have resulted in higher IFX trough concentrations that potentially masked ADA detection by GRA or immunoassay in these patients [30]. Even though the immunoassay we used included an acid-dissociation step that significantly increases the drug tolerance of the assay, drug tolerance is one inherent limitation of studies examining ADAs [31]. An additional explanation for the lower incidence of IFX ADAs in patients with JIA and uveitis may be related to the higher rate of concomitant immunomodulator therapy use in this population (75% in JIA/uveitis vs. 30% in IBD); although, in our current cohort, concomitant medications did not appear to influence IFX clearance or the development of ADAs.

Consistent with previous studies, our data support findings of increased IFX clearance in patients with lower serum albumin concentrations, elevated ESRs, and patients positive for IFX ADAs [16, 17]. However, we failed to demonstrate a significant relationship between IFX clearance and other previously investigated covariates, such as diagnosis, age, weight, and DMARD use. By covariate analysis using population pharmacokinetic modeling, we were able to develop a model to describe the relationship between IFX clearance for individuals and clinical covariates including ADA positivity by GRA, serum albumin levels and ESR. In contrast to previous work, weight and DMARD use were not shown as significant covariates for IFX clearance in our data [21]. We did find that IFX troughs were significantly higher in patients receiving combination therapy with methotrexate than monotherapy with IFX (21.2 [12.3,39.5] vs 13.1 [5.9,25.2] μg/mL, *p* = 0.01), however, patients receiving combination therapy were also receiving higher cumulative doses of IFX. Once dosing differences were accounted for in the pharmacokinetic modeling, no detectable effect of MTX on IFX pharmacokinetics was observed. This is contrary to observations of decreased IFX clearance with combination therapy in a post hoc pooled population analysis of the REACH trial of IFX in pediatric IBD [21]. This discrepancy may reflect the significantly higher IFX dose (median [IQR]: 8.2 [6.4,9.7] mg/kg) and shorter dosing interval (median [IQR]: 6 [4,7] weeks) used in our study compared to the REACH trial (5 mg/kg every 8-12 weeks). In particular, early studies investigating the impact of MTX on reducing IFX clearance demonstrated the greatest impact at extremely low doses of IFX (i.e. 1 mg/kg), with significantly reduced effects as IFX doses were increased [32]. Other studies have commented on observations of higher IFX troughs in patients receiving combination therapy with azathioprine or 6-mercaptopurine [33]. No significant differences were observed in our study; however, this may be due to the small number of patients receiving concomitant therapy with azathioprine (*n* = 3).

In a separate analysis we evaluated whether clinical disease activity in IBD and JIA were associated with increased IFX clearance. Such an analysis could not be performed for uveitis, as standardized disease activity scoring data were lacking for these patients. Although we had a limited number of patients with JIA with corresponding clinical disease activity scores, a significant positive correlation between cJADAS-71 and IFX clearance was observed. Specifically, when stratified by a low disease activity cJADAS-71 cut-off score of 2.5 that could be applied to both oligoarthritis and polyarthritis, patients with active disease had estimated IFX clearance values 49% higher than patients with low disease activity. This suggests that these patients would require higher IFX doses to achieve a similar level of exposure, indicating that these patients may be hypermetabolic. A similar trend was observed in our IBD cohort, with IFX clearance 50% higher in children with moderate/severe vs. quiescent disease, and 47% higher in moderate/severe vs. mild disease; however this trend did not reach statistical significance (*p* ≤ 0.08), likely due to limitations in sample size for moderate/severe IBD (*n* = 8). There may also be a potential limitation in the clinical assessment used for disease activity in the IBD population. In our study, we used the readily and consistently available PGA score, a compilation of clinically meaningful signs and symptoms in addition to objective laboratory values, rather than endoscopy, which is invasive and not always clinically indicated or performed to assess treatment response or mucosal healing. Nevertheless, there remained an association of increased IFX clearance with elevated ESR and low albumin levels, both physiologic markers of inflammatory burden. Together, these data support a potential relationship between increased disease burden and enhanced IFX clearance and suggest that IFX dosing intensity may need to be increased in the presence of active disease and continued elevations in inflammatory markers.

Employing therapeutic drug monitoring (TDM) as an individualized treatment strategy to guide IFX dosing has been shown to optimize efficacy, safety and cost effectiveness of biologic agents such as IFX in IBD [34–36], and a trial is currently underway to determine the effectiveness of standardized TDM in IFX management across different diagnoses in adults [37]. Our data suggest that dose individualization for children may need to go well beyond standard pediatric dosing practices in IBD (e.g., 5 mg/kg every 8 weeks), and perhaps clinical features such as markers of disease activity could judiciously guide higher dosing in children with rheumatic disease. However, future prospective longitudinal studies will be necessary to further delineate the relationship between disease activity and dose intensity, as the risk for under dosing children with IFX is unquestionable when disease activity is inadequately treated and long-term morbidity is at stake. Future studies will also need to further delineate the relationship between drug toxicity and dose intensity to begin to weigh the risks of increased or excessive exposure to IFX. Identification of potential upper thresholds of exposure may be able to minimize IFX related toxicities, such as excessive immunosuppression and risk for infection, or simply identify dosing that exceeds additional benefit to maximize cost effectiveness. In the realm of pediatric rheumatic disease, there are significant gains to be made in this domain.

## Conclusions

Overall, our data demonstrate no differences in IFX pharmacokinetics among children with different autoimmune disorders; however, several factors impact drug exposure. In this real-world clinical cohort, children with IBD had lower IFX troughs and higher prevalence of IFX ADAs (12%) than children with uveitis or JIA (0%). Over 20% of children with IBD had trough concentrations in ranges that were previously established as inadequate for mucosal healing [24]. Although children with JIA and uveitis are not utilizing therapeutic drug monitoring with IFX trough targets yet, our findings support that increased IFX doses and/or decreased dosing intervals positively impacts drug trough levels and may be critically important in patients with an elevated inflammatory burden where systemic IFX clearance appears to be significantly increased. Understanding how drug trough levels correlate with drug response in JIA and uveitis will be important next steps to further a targeted approach to treatment with IFX in pediatric rheumatic disease.

## Supplementary Information

**Additional file 1.**

## Data Availability

The datasets used and/or analyzed during the current study are available from the corresponding author on reasonable request.

## Abbreviations

ADAs: Anti-drug antibodies
AZA: Azathioprine
cJADAS-71: 71-Joint count clinical juvenile arthritis disease activity score
Cl: Clearance
CRP: C-Reactive protein
DMARD: Disease modifying anti-rheumatic drug
ELISA: Enzyme-linked immunosorbent assay
ESR: Erythrocyte sedimentation rate
GRA: Gene-reporter assay
IBD: Inflammatory bowel disease
INF: Infliximab
JIA: Juvenile idiopathic arthritis
MTX: Methotrexate
OFV: Objective function value
PGA: Physician global assessment
Q: Intercompartmental clearance
TDM: Therapeutic drug monitoring
TNFα: Tumor necrosis factor-alpha
V1: Volume of the central compartment
V2: Volume of the peripheral compartment
