# Supplementary material for: Factors associated with reduced infliximab exposure in the treatment of pediatric autoimmune disorders: a cross-sectional prospective convenience sampling study
Source: Pediatr Rheumatol Online J. 2021 May 1;19:62. doi: 10.1186/s12969-021-00548-8 (PMC8088679; doi:10.1186/s12969-021-00548-8)
Supplement: Supplementary file 1 — Additional file 1. [file 12969_2021_548_MOESM1_ESM.docx]

**Supplemental Data**

**Supplementary Table 1.** Patient clinical characteristics in population covariate modeling.

| **Categorical covariate** | | **Number of patients in category** |
| --- | --- | --- |
|  | Diagnosis (IBD/JIA/Uveitis) | 72/8/2 |
|  | Gender (Male/Female) | 48/34 |
|  | DMARD (N/Y) | 52/30 |
|  | MTX (N/Y) | 55/27 |
|  | AZA (N/Y) | 79/3 |
|  | IFX ADA by GRA (N/Y) | 79/3 |
|  | IFN ADA by ELISA (N/Y) | 74/8 |
| **Continuous covariate** | | **Mean ± standard deviation or median [range]** |
|  | Age (yr) | 15 ± 3.7 |
|  | Weight (kg) | 60.3 ± 21.2 |
|  | Albumin (g/dL) | 4.3 [3.1,5.1] |
|  | CRP (mg/dL) | 0.5 [0,14.2] |
|  | ESR (mm/hr) | 9 [2,61] |

**Supplementary Table 2.** Stepwise selection of covariates in IFX population pharmacokinetic model.

| **Model** | **ΔOFV** | **p value** |
| --- | --- | --- |
| **Forward addition** |  |  |
| 1. Base model | 0 | - |
| 1. 1 + GRA | -20.58 | <0.0001 |
| 1. 2 + Albumin | -39.63 | <0.0001 |
| 1. 3 + ESR | -48.53 | <0.0001 |
| **Backward deletion** |  |  |
| 1. Full model | 0 | - |
| 1. 1 – GRA | 25.27 | <0.0001 |
| 1. 1 – Albumin | 13.68 | <0.001 |
| 1. 1 – ESR | 8.9 | <0.005 |

**Supplementary Table 3.** Population pharmacokinetic parameter estimates of the final covariate model in 82 patients where: Cl_ind_ = Cl_pop_ X (albumin/4.3)^-1.8^ X (ESR/9)^0.069^ X 1.04^GRA^ X exp(η_cl_). Cl_ind_ = individual estimate of IFX clearance, Cl_pop_ = population estimate of IFX clearance, GRA=1 in patients observed ADAs by GRA and GRA=0 in patients with no observed ADAs by GRA. The η_cl_ represents the interindividual variability of IFX clearance. Albumin and ESR were centered by median value of the covariate in the population. The final model OFV was reduced by 51.59 compared to the base model.

|  | **Parameters** | **Estimate** | **Relative standard error (%)** |
| --- | --- | --- | --- |
| **Fixed Effects** | | | |
|  | Cl (L/kg/d) | 0.00231 | 47.2 |
|  | V_1_ (L/kg) | 0.0542 (FIX) |  |
|  | Q (L/kg/d) | 0.00352 (FIX) |  |
|  | V_2_ (L/kg) | 0.0292 (FIX) |  |
|  | GRA on Cl | 1.04 | 18.2 |
|  | ALB on Cl | -1.8 | 25.3 |
|  | ESR on Cl | 0.0687 | 39.6 |
| **Standard Deviation of the Random Effects** | | | |
|  | Interindividual variability of Cl | 0.293 | 12.6 |
| **Error Model Parameters** | | | |
|  | Additive term | 0.328 | 56.9 |

**Supplementary Figure 1.** Evaluation of the final covariate model. **(A)** Observed concentrations versus individual predicted concentrations and **(B)** individual predicted residuals versus time or IFX concentration (Cc) were shown as part of goodness-of-fit.

**
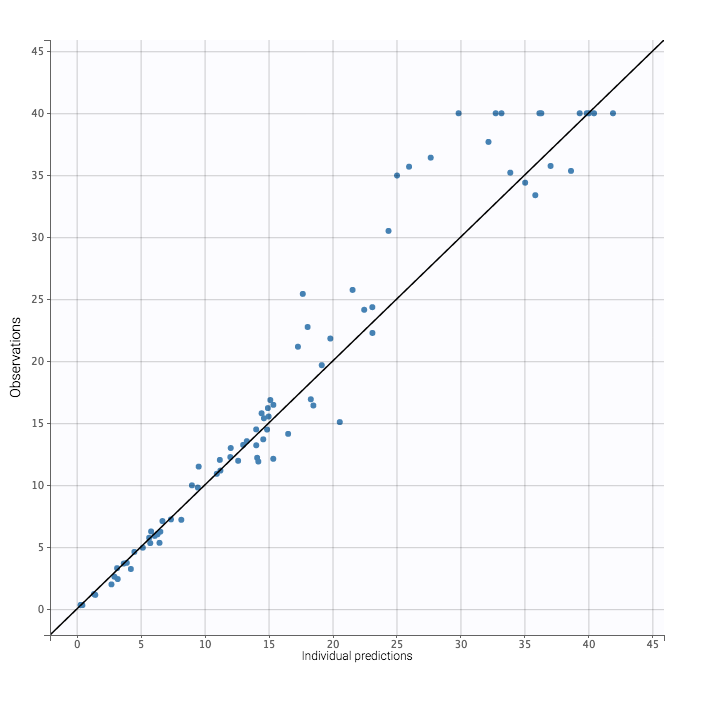
(A)**

**
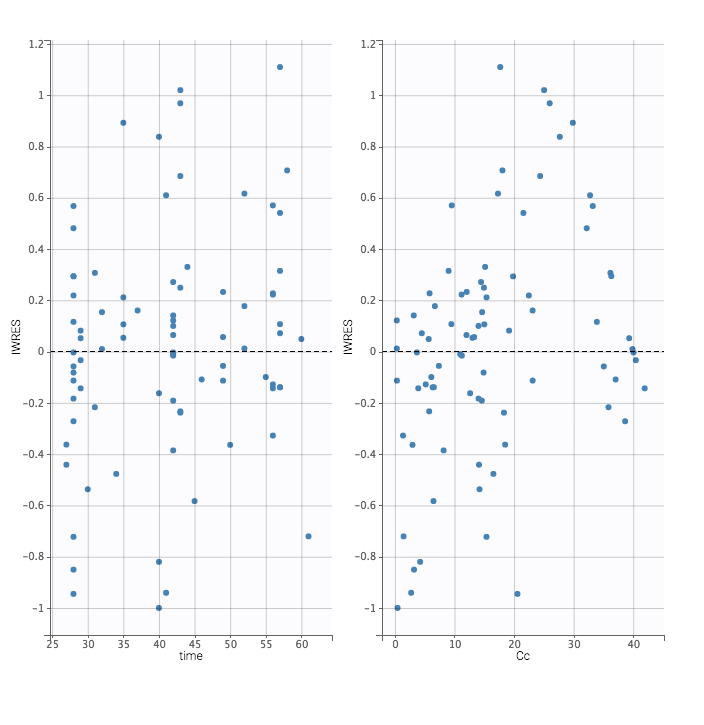
(B)**
